# Perceived stress, reasons for and sources of stress among medical students at Rabigh Medical College, King Abdulaziz University, Jeddah, Saudi Arabia

**DOI:** 10.1186/s12909-018-1133-2

**Published:** 2018-02-23

**Authors:** Zohair Jamil Gazzaz, Mukhtiar Baig, Bader Salem Mana Al Alhendi, Mohammed Mahdi Owiad Al Suliman, Awshaemah Salem Al Alhendi, Mana Saleh Hadi Al-Grad, Mohammed Abdullah Ali Qurayshah

**Affiliations:** 1 Department of Medicine, Faculty of Medicine, Rabigh, King Abdulaziz University, Jeddah, 21589 Saudi Arabia; 2 Department of Clinical Biochemistry/Medical Education, Faculty of Medicine, Rabigh, King Abdulaziz University, Jeddah, 21589 Saudi Arabia; 3 Students, Faculty of Medicine, Rabigh, King Abdulaziz University, Jeddah, Saudi Arabia

**Keywords:** Perceived stress, Sources of stress, Medical students, Jeddah, Saudi Arabia

## Abstract

**Background:**

Medical students have high levels of stress that could be due to the daily life stressors and the extra stress of academic burden. The present study investigated the perceived stress level as well as the reasons and sources of stress among medical students at a comparatively newly established medical college affiliated with King Abdulaziz University (KAU), Jeddah, Saudi Arabia (SA).

**Methods:**

The present study was carried out at Rabigh Medical College (RMC), KAU, Jeddah, and completed in 2015. The data was collected by an anonymous self-administered questionnaire that has three components: a list of 33 items of probable stressors, perceived stress scale, and demographic information and academics.

**Results:**

The response rate in our study was 86% (152/176), the mean age was 20.35 ± 1.09, 77 (51%) were from preclinical years and 75 (49%) from clinical years. The mean PSS score among our participants was 28.5 ± 3.8 with a median of 28.0 (IQR 26.0–31.0) and 59.2% of participants were stressed. The mean PSS score 30.6 ± 4.4 for students with sibling > 5 was significantly higher as compared to the mean PSS score (27.9 ± 3.3) for students with sibling ≤5. Similarly, the mean PSS score (32.0 ± 3.4) of students with often/always occurrence of psychosocial stressors was higher as compared to the mean PSS score (28.3 ± 3.7) of those students with less than a frequent occurrence of stressors. Moreover, those students that were more stressed had lower marks in the last exam (< 80%) as compared to students with less stress who had higher marks (≥80%) (*P* < .05).

Performance in practicals, examinations frequency, disappointment with the class lectures, lack of personal interest in medicine, lengthy academic curriculum/syllabus, worries about the future and periodic examinations performance were rated as severe.

The logistic regression analysis showed that stress cases were linked with last exam marks [OR 1.26, 95% CI 0.64–2.48], number of siblings [OR 2.27, 95% CI 0.97–5.27], and academic stressor [OR 2.02, 95% CI 0.61–6.66] but no significant relationship was found.

**Conclusion:**

There were high-stress levels among the participants of this study, and the main stressors were academic-related.

## Background

Medical students come across numerous anxieties during their transformation from novice student to an expert and well-informed physician. There is a growing body of evidence that because of extensive research in the medical sciences there is a rapid expansion in medical knowledge that medical students are believed to understand and be able to use in different circumstances in their professional life [1].

Generally, the medical students have high levels of stress that could be due to the daily life stressors and the extra stress of academic burden, lack of relaxation time, breadth and depth of material to be learned, and repeated formative and summative examinations in a competitive environment [2]. A recent study reported stress (62%) and burnout (75%) among preclinical medical students [3]. Stress and burnout among medical students is a common problem with likely severe personal and professional effects [4].

The milieu of medical education is considered as difficult and time-demanding, and more commitment and dedication are needed. Globally, numerous studies have reported stress among undergraduate medical students to be 25.6% - 78% [3, 5, 6].

A minor level of stress is beneficial and enables the student to become a more dynamic and better performer. Conversely, persistently high levels of stress may cause considerable psychological and physical glitches like poor academic performance, stress-related anxiety, depression, drug use, and even suicide [7–9]. Being a medical educator, it is imperative to identify stress and reasons for stress among students because it has adverse effects on the students’ health, their academic performance, and on their career achievements [2, 10].

There are various types of stressors, which have a negative effect on mental capabilities, the learning process, and academic progress. Previous reports have categorized stressors into three major groups: academic, psychosocial and health-related [11, 12]. Several earlier researches have documented the role of academics, gender, marital status, and age as potential stressors [5, 11, 13, 14].

The enrolled students at the RMC, Faculty of Medicine, KAU, Jeddah, belonged to different urban and rural areas of the Kingdom. Therefore, these admitted students have varied customs, educational backgrounds and socioeconomic conditions. Most of the students get their early education in Arabic-medium schools; then, suddenly they are exposed to an entirely different instructional and learning environment where all lectures and subjects are in English and they have to take examinations in English as well. These factors may collectively affect most of the students’ performance, and they may feel stressed, which may affect their academic performance.

Several studies conducted in Arab countries, such as Egypt (60%) [15], Sudan (50%) [8], Lebanon (62%) [3], and few studies in Saudi Arabia (72%) [16], (53%) [17], and (63%) [18], showed high levels of stress among medical students.

However, this is the first study in our newly established medical college that measures the perceived stress level and potential stressors among undergraduate medical students. The present study investigated the perceived stress level as well as reasons and sources of stress among medical students at the RMC, Faculty of Medicine, Rabigh, KAU, Jeddah. Our results would be significant enough for instructors and institutional policymakers to adopt strategies that may help in alleviating modifiable reasons of stress and stressors.

## Methods

The present cross-sectional study was carried out at RMC, Faculty of Medicine, KAU, Jeddah, and completed in 2015. The Medical College in Rabigh is comparatively new in the Kingdom of Saudi Arabia and up till now three batches have passed out. Rabigh is a small town located in the coastal area of the Red Sea with the population less than two hundred thousand [19]. At RMC, there is a modular system and after each module there is a summative assessment and, during module one to two, assessment tests are held according to the length of the module. All modules of less than six weeks have one mid-module test and those modules having > 6 weeks have two tests before the final exam. The written exam is comprised of MCQs and short answer questions (SAQs). For preclinical years there is the objective structured practical exam (OSPE), and for clinical years there is the objective structured clinical exam (OSCE). In a few clinical modules, short and long cases are also used. So there are multiple tools of assessment being used at RMC.

There are only male students in the RMC. In each class there are 20–45 students and we encouraged all 176 students to participate in this research. In the initial batches there were about 20, so we also included students of the batch who had recently passed out. The sample size for our study was 121. It was calculated by the OpenEpi Version 3 open source calculator, taking the prevalence of stress among medical students as 53% [17], confidence level as 95%, population size as 176, and power of the study as 80%.

The data was collected by an anonymous self-administered bilingual questionnaire (English and Arabic languages) and the participants were selected from each class by convenience sampling method. In this study, only students of Rabigh Medical College were included, and we did not include the students of Faculty of Medicine, Jeddah, KAU or from any other medical college.

The potential stressors given in the study questionnaire were taken from previously published studies [11, 12]. There are 33 stressors, which are classified as academic, health-related and psychosocial. For each potential stressor the frequency of occurrence was classified as never, rarely, sometimes, often and always, and these were scored as 1, 2, 3, 4 and 5, respectively. Each stressor had its severity rated using a Likert scale (1–10) ranging from not severe to very severe. The students were required to indicate if they had been affected by any of the stressors.

The principal investigator translated the English version into Arabic with the help of co-researchers. It was further checked and modified by the two senior bilingual faculty members (Arabic and English). This bilingual questionnaire (English and Arabic languages) was then pretested on a group of 50 students. Comprehension and internal consistency of this questionnaire were found by Cronbach’s alpha to be .86.

Data was also gathered on age, marital status, and marks in the last examination, with percentages as to whether living in hostel or day scholar, number of siblings, father’s annual income, and hobbies.

We used the already validated and reliable Perceived Stress Scale (PSS-14) for measuring perceived stress. The reliability of PSS is 0.85 (Cronbach’s coefficient) with a test-retest reliability during a short retest interval (several days) of 0.85 [20]. The PSS-14 was translated into the Arabic language and this bilingual questionnaire was used to collect the data. In Saudi Arabia, the medium of teaching in medical colleges is English; therefore, the researchers assumed that the students can understand the questionnaire in English, but to improve its comprehension each question was translated into Arabic as well. Moreover, to know the language and understanding of the questionnaire, it was pretested on 50 students, and the internal consistency of this questionnaire was calculated by Cronbach’s alpha to be .87.

“The PSS does not tie appraisal to a particular situation; it is sensitive to the non-occurrence of events as well as to ongoing life circumstances” [21]. “The stress score was stratified into no, mild, moderate stress (less than the first, second and third quartiles, respectively) (merged as low level) or severe stress (equal to or above the fourth quartile) (high level)” [21].

Among the fourteen items of PSS-14, there are seven positive ones (4, 5, 6, 7, 9, 10, 13) and seven negative ones (1, 2, 3, 8, 11, 12, 14), representing perceived self-efficacy and helplessness, respectively. Each item was rated on a five-point Likert-type scale (0 = never to 4 = very often). After reversing positive items’ scores and then summing up all scores, the total scores were computed. The range for total scores for PSS-14 is from 0 to 56. This scale has numerical values and a higher value points out more stress and a lower value signifies low stress. In our study, the prevalence of stress was found to be 59% by taking the 28 PSS score as the cut-off value between the stressed and the unstressed students, based on the quartiles. However, “the Perceived Stress Scale is not a diagnostic instrument; there are no score cut-offs. There are only comparisons within your own sample” [20].

We also measured the academic progress of the participants by their performance in the last module examination. Furthermore, the study was carried out in the middle of the module to avoid examination stress as a confounding factor. The objective of the study was explained to all participants and strict confidentiality was maintained. The Ethical Review Committee of the Faculty of Medicine, Rabigh, granted the Ethical approval for this study.

### Statistical analysis

The data were analyzed using SPSS (Statistical Package for Social Sciences) version 23. Frequency and percentage were given for each statement of Perceived Stress Scale (PSS) and sources of stress. Perceived severity was given in median with interquartile range. Mean ± SD was given for quantitative variables, i.e., PSS. The Shapiro–Wilk test was used to check the normality of data. Data was normally distributed, so independent sample t-test and one-way ANOVA test were used to observe the mean difference in PSS between demographic variables and groups of stressors. Univariate and multivariate logistic regression were applied to evaluate causal factors of stressed cases considering perceived stress (stressed cases) as the dependent variable, groups of stressors (i.e. academic, psychosocial and health-related) and demographic variables as the independent variables. Univariate and adjusted odds ratios (OR) with 95% confidence intervals (95% CI) were computed. The level of *p*-value < 0.05 was taken as significant.

## Results

The response rate in our study was 86% (152/176); the mean age was 20.35 ± 1.09, and 77 (51%) were from preclinical years and 75 (49%) from clinical years. All students were Saudi nationals, 140 were unmarried, 12 were married; 124 came from the urban areas and 28 from the rural areas. Fifty-four students (35%) had 80% marks in the last exam while 98 (65%) had < 80% marks. There were 118 (78%) students who had ≤5 siblings while 34 (22%) students had > 5 siblings. One hundred and twenty-five (82%) students were day scholars while 27 (18%) were residing in the hostels (Boarders). The students’ response to the perceived stress scale is presented in Table 1.

**Table 1:** Students response to the perceived stress scale

Statement	Never N(%)	Almost never N(%)	Some-times N(%)	Often N(%)	Very often N(%)
In the last month, how often have you been upset because of something that happened unexpectedly?	8 (5.3)	21 (13.8)	57 (37.5)	43 (28.3)	23 (15.1)
In the last month, how often have you felt that you were unable to control the important things in your life?	23 (15.1)	38 (25.0)	50 (32.9)	25 (16.4)	16 (10.5)
In the last month, how often have you felt nervous and “stressed”?	13 (8.6)	13 (8.6)	31 (20.4)	59 (38.8)	36 (23.7)
In the last month, how often have you dealt successfully with day to day problems and annoyances?	25 (16.4)	32 (21.1)	54 (35.5)	31 (20.4)	10 (6.6)
In the last month, how often have you felt that you were effectively coping with important changes that were occurring in your life?	15 (9.9)	40 (26.3)	49 (32.2)	33 (21.7)	15 (9.9)
In the last month, how often have you felt confident about your ability to handle your personal problems?	14 (9.2)	37 (24.3)	49 (32.2)	32 (21.1)	20 (13.2)
In the last month, how often have you felt that things were going your way?	14 (9.2)	33 (21.7)	53 (34.9)	35 (23.0)	17 (11.2)
In the last month, how often have you found that you could not cope with all the things that you had to do?	7 (4.6)	31 (20.4)	53 (34.9)	43 (28.3)	18 (11.8)
In the last month, how often have you been able to control irritations in your life?	25 (16.4)	32 (21.1)	55 (36.2)	28 (18.4)	12 (7.9)
In the last month, how often have you felt that you were on top of things?	16 (10.5)	33 (21.7)	29 (19.1)	52 (34.2)	22 (14.5)
In the last month, how often have you been angered because of things that happened that were outside of your control?	35 (23.0)	29 (19.1)	64 (42.1)	20 (13.2)	4 (2.6)
In the last month, how often have you found yourself thinking about things that you have to accomplish?	12 (7.9)	38 (25.0)	45 (29.6)	37 (24.3)	20 (13.2)
In the last month, how often have you been able to control the way you spend your time?	14 (9.2)	29 (19.1)	55 (36.2)	32 (21.1)	22 (14.5)
In the last month, how often have you felt difficulties were piling up so high that you could not overcome them?	14 (9.2)	42 (27.6)	44 (28.9)	32 (21.1)	20 (13.2)

The respondent sources of stress described as frequent/constant were frequency of examinations [92(60%)], performance in practicals [78(51%)], lack of personal interest in medicine [60(39%)], difficulty in the journey back home [71(47%)], lengthy academic curriculum [84(55%)], periodic examinations performance [64(42%)], worries about the future [72(47%)], becoming a doctor [67(44%)], and competition with peers 60(39%). Examination frequency, performance in practicals, disappointment with the class lectures, lengthy academic curriculum/syllabus, worries about the future and periodic examinations performance was rated as severe (Table 2).

**Table 2:** Students response to several sources of stress and perceived severity (rated in a Likert scale of 1–10) as reported by the students)

Sources of stress	Frequency of occurrence			Severity	
	Never /Rarely	Some-times	Often/Always	Median	IQR*
a) Academic sources					
Frequency of examination	30	30	92	6	4–7
Performance in Examinations	51	37	64	5	3–7
Academic Curriculum	40	28	84	5	3–6
Dissatisfaction with class lectures	51	48	53	5	3–7
Non-availability of adequate learning materials	58	36	58	5	2–6
Becoming a Doctor	58	27	67	3	2–4
Lack of time for recreation	62	38	52	3	2–4
Competition with Peers	46	46	60	3	2–5
Performance in practicals	38	36	78	3	2–4
Lack of special guidance from faculty	68	36	48	2	2–4
b) Psychosocial stressors					
High Parental Expectations	61	37	54	4	2–6
Loneliness	52	42	58	5	3–6
Family Problems	73	36	43	2	1–4
Accommodation away from home	88	46	18	2	1–4
Political situation in the country	52	41	59	1	1–1
Worrying about the future	49	31	72	5	3–6
Relations with the Opposite Sex	75	38	39	1	2–4
Difficulty in reading text books	56	54	42	3	4–5
Lack of entertainment in the institution and the city	55	51	46	2	3–4
Difficulty in the journey back home	26	55	71	2	3–4
Financial strain	90	36	26	2	3–4
Inability to socialize with peers	41	52	59	3	1–5
Living conditions in the hostel	140	05	07	2	1–4
Member of fraternity or sorority	58	47	47	2	1–4
Lack of personal interest in medicine	64	28	60	3	1–4
Adjustment with roommate/s	132	07	13	1	1–4
c) Health related stressors					
Sleeping Difficulties	85	29	38	4	2–6
Class Attendance	73	58	21	3	2–4
Nutrition	44	31	77	4	2–4
Exercise	80	45	27	4	2–4
Quality of food in mess	134	07	11	2	1–3
Physical disability	82	23	47	1	1–2
Alcohol/Drugabuse/Smoking	57	40	55	1	1–4

*Inter Quartile Range

The students’ response to PSS is shown in Table 3. The mean PSS score in the study population was 28.5 ± 3.8 with a median of 28.0 (IQR 26.0–31.0). In our study 59.2% of the participants were stressed. The mean PSS score of 30.6 ± 4.4 for students with sibling > 5 was significantly higher as compared to the mean PSS score (27.9 ± 3.3) for students with sibling ≤5. Similarly, the mean PSS score (32.0 ± 3.4) for students with frequent/always occurrence of psychosocial stressors was higher as compared to the mean PSS score (28.3 ± 3.7) for those students with less than a frequent occurrence of stressors. Moreover, those students with more stress had lower marks in the last exam (< 80%) as compared to students with less stress who had higher marks (≥80%) (*P* < .05). No significant difference in mean PSS was found among different demographic variables and groups of stressors (Table 3).

**Table 3:** Comparison of PSS among different factors

Determinants	N	Mean	SD	*p*-value
Overall PSS	152	28.5	3.8	–
Year of study				
Pre clinical	77	28.7	3.6	0.453
Clinical	75	28.3	3.9	
Marital Status				
Unmarried	140	28.5	3.8	0.586
Married	12	29.1	3.9	
Living in				
Day scholar	125	28.7	3.8	0.264
Boarding	27	27.8	3.3	
Residence				
Urban	124	28.6	4.0	0.459
Rural	28	28.0	2.8	
Father Income				
> 20,000	64	29.2	4.2	0.128
10,000–20,000	59	28.1	2.9	
< 10,000	29	27.8	4.2	
Last exam mark				
≥ 80	54	27.7	3.3	0.060
< 80	98	28.9	3.9	
Number of sibling				
≤ 5	118	27.9	3.3	0.002*
> 5	34	30.6	4.4	
Occurrence of Academic Stressors				
Less than often	137	28.4	3.8	0.458
Often/Always	15	29.2	3.3	
Occurrence of Psychosocial Stressors				
Less than often	145	28.3	3.7	0.012*
Often/Always	7	32.0	3.4	
Occurrence of Health Stressors				
Less than often	143	28.4	3.8	0.345
Often/Always	9	29.7	3.5	

*Significant

The logistic regression analysis showed that stress cases were linked with last exam marks [OR 1.26, 95% CI 0.64–2.48], number of siblings [OR 2.27, 95% CI 0.97–5.27], academic stressor [OR 2.02, 95% CI 0.61–6.66] but no significant relationship was found (Table 4).

**Table 4:** Determinants of stress cases by logistic regression

Determinants	No.	No. of stressed	Univariate OR (95% CI)	Adjusted OR* (95% CI)
Year of study				
Pre clinical	77	50 (64.9)	1	1
Clinical	75	40 (53.3)	0.62(0.32–1.18)	0.53(0.25–1.10)
Marital Status				
Unmarried	140	84 (60.0)	1	1
Married	12	6 (50.0)	0.67(0.21–2.17)	0.85(0.24–3.06)
Living in				
Day scholar	125	75 (60.0)	1	1
Boarding	27	15 (55.6)	0.83(0.36–1.93)	0.93(0.24–2.54)
Residence				
Urban	124	73 (58.9)	1	1
Rural	28	17 (60.7)	1.08(0.47–2.50)	0.98(0.38–2.52)
Father Income				
> 20,000	64	45 (70.3)	1	1
10,000–20,000	59	29 (49.2)	0.41(0.19–0.86)	0.43(0.19–0.97)
< 10,000	29	16 (55.2)	0.52(0.21–1.29)	0.68(0.25–1.87)
Last exam mark				
≥ 80	54	30 (55.6)	1	1
< 80	98	60 (61.2)	1.26(0.64–2.48)	1.11(0.52–2.39)
Number of sibling				
≤ 5	118	65 (55.1)	1	1
> 5	34	25 (73.5)	2.27(0.97–5.27)	2.16(0.84–5.57)
Occurrence of Academic Stressors				
Less than often	137	79 (57.7)	1	1
Often/Always	15	11 (73.3)	2.02(0.61–6.66)	1.95(0.477.99)
Occurrence of Psychosocial Stressors				
Less than often	145	83 (57.2)	–	–
Often/Always	7	7 (100.0)		
Occurrence of Health Stressors				
Less than often	143	85 (59.4)	1	1
Often/Always	9	5 (55.6)	0.85(0.22–3.31)	0.91(0.20–4.22)

*Odds ratio

## Discussion

The mean PSS score of our participants was 28.5 ± 3.8. In our study more than half (59.2%) of the participants were stressed. Our results show that stress prevalence among medical students in our college is high. This is similar to studies from Pakistan [2], Portugal [22], Saudi Arabia [23] and Trinidad and Tobago [24].

In our study participants, the mean PSS score for students with siblings > 5 was significantly higher as compared to the mean PSS score for students with sibling ≤5. Similarly, the mean PSS score for students with frequent/always occurrence of psychosocial stressors was higher as compared to mean PSS score for those students with less than a frequent occurrence of stressors. Moreover, those students that were more stressed had lower marks in the last exam (< 80%) as compared to students with less stress who had higher marks (≥80%). However, our study could not find significant differences in mean PSS scores between preclinical and clinical students, married and unmarried students, day scholar and boarding, residence in urban and rural areas, father’s income, the existence of academic stressors, and the existence of health stressors. No significant differences in mean PSS were found among different demographic variables and groups of stressors. The reasons for these could be that, in the KSA, education and health facilities are free for all Saudi nationals. Moreover, all college students including medical students get a good amount of money as monthly stipend from the Ministry of Higher Education and the university also provides them hostel residence and food on nominal charges.

Our results are similar to Amr et al.’s (2009) [21]. They reported no significant differences in the prevalence of stress between preclinical and clinical classes, urban and rural groups, and family income. A Malaysian study reported no significant difference in prevalence of stress between clinical and preclinical students [25]. A study from Pakistan stated that students failing in their exams had high-stress levels [2]. In contrast to our results, few recent studies found a noteworthy difference in the perceived stress levels among day scholars and residents [5] or higher-level stress among clinical year students as compared to preclinical years [24]. Reasons for the dissimilarity of our study results to other studies could include the fact that only male participants were included in our study, cultural variances, the difference in the educational environments, type of instrument used for measuring stress, the difference in the population characteristics. Moreover, our college is newly established.

In the current study, academic stressors were more common among our participants. These results are in concordance with a few other studies [5, 11, 12]. In contrast to our results, a study reported that in addition to educational demands social and physical factors were major reasons for the psychological disturbance among students [26]. A study observed that both psychosocial and academic issues were common to the cohort [14]. In most of the Kingdom’s public schools, the medium of education is Arabic, but in medical education the medium of teaching is English and all curriculum is in English, so the language barrier could be one of the reasons for their stress.

Among our study participants, frequency of examinations, performance in practicals, unhappiness with the class lectures, less personal interest in medicine, lengthy academic curriculum/syllabus, worrying about the future and periodic examinations performance were rated as common stressors. Our results are by and large similar to several other studies [5, 11, 13, 14].

In a recent study in Saudi Arabia, the students perceived the exams, course load and hectic timetable as the major reasons for their depression, anxiety and stress [23]. A study in India reported that academic and physical reasons were found to be the main sources of stress [26].

In the present study, the logistic regression analysis showed that stress cases were linked with last exam marks (< 80%), number of siblings (> 5) and academic stressors (often/always), but no significant relationship was found. A study reported a negative significant correlation with academic performance and sources of perceived stress and stress levels [2] while another study reported a link between stress cases and academic and psychosocial stressors [11]. The presence of stress among low achievers is natural, as lower marks tell on their self-esteem and make them feel inferior among their classmates.

Neglecting one’s psychological problem could have to grave consequences [27]. Dyrbye et al. (2010) reported that burnout and chronic stress strongly correlate with professional misbehavior or decreased altruistic values among doctors [28], and it seems a grave problem not only for the physicians but also for the society. We suggest that students should adopt stress coping strategies and students’ counselors should provide guidance about stress coping strategies.

Our study and a few other studies have pointed out frequency of examination as an important stressor; therefore, there is a need to improve students’ assessment process and to make it less stressful and student-friendly [13]. Moreover, students should be supported to take part in sports and other extracurricular activities that can alleviate stress, anxiety and burnout as well as their consequences on physical and mental health [13, 29]. A study suggested student-led support programs designed to promote mentorship of newly admitted junior students by senior students to help them acclimatize to the new medical school setting [3]. Recently, a study in Egypt reported a significant association between obesity and stress and higher stress and anxiety scores were found among obese and overweight medical students [30]. A research group recently reported that the lifestyle and dietary habits of the majority of the students at Rabigh campus were not up to the mark and the prevalence of obesity was common [31]. Another study reported that 49% of KAU students were either overweight or obese and 7% were hypertensive and the lifestyle of the majority of the students was not healthy [32]. Therefore, one may assume that physical inactivity and obesity among students could be one of the causes of stress.

A recent study reported an important and interesting finding that students who practiced a faith and considered their religion important had lower levels of depression and burnout [20]. Another study from India has pointed out that religion and acceptance were the most important stress coping strategies [33]. Therefore, following religious principles could possibly be a good coping mechanism.

Students need to know the prevention strategies for coping with stressful situations. It is suggested that universities and colleges should aim to reduce stressors and to provide psychosocial and academic support systems to alleviate students’ stress. Early detection can play a very important role in this regard.

It should be a cause of concern for our university administration that our students have high stress levels, and it calls for a remedy to lessen the levels of stress among medical students. There is an urgent need to address this matter by the related institute through the provision of a conducive educational environment that would have a positive impact on learning.

This study has a few limitations. Firstly, it is a questionnaire-based study; so reporting bias cannot be ignored. Secondly, it is a single-center study in a newly established medical college so our results cannot be generalized, and it is quite possible that students of other medical colleges in Saudi Arabia have different types of stressors. Thirdly, our study participants are only male because at the Faculty of Medicine Rabigh there are only male students.

## Conclusion

There were high stress levels among the participants of this study, and performance in practicals, frequency of examinations, unhappiness with the class lectures, lengthy academic curriculum/syllabus, worrying about the future and periodic examinations performance were rated as common stressors. The academic stressors were more common among our participants. Therefore, it is suggested that frequent assessment should be avoided, the teaching and learning environment should be conducive, and there should be a focus on active teaching and learning activities. We believe that students’ active involvement in the educational process would help in reducing the academic stressors.

There is a need for early detection and different intervention measures should be taken to reduce stressors, and in this context proper counseling and academic support may play an important role. Chronic stress may affect professional attitude and the resulting poor performance would ultimately have an impact on the society. We have few suggestions such as the establishment of a students’ counseling body, increase in sports activities and other extracurricular activities in the college. Moreover, the students should be made aware of stress coping strategies, the importance of a healthy lifestyle pattern, and physical activities. There is a need for longitudinal studies to recognize different sources and reasons for stress among students of all the medical colleges in the KSA, and dedicated and collaborative efforts are needed from the authorities to deal with this serious problem.

It is recommended that there should be the use of depression, anxiety, and stress-measuring inventories at the time of admission into the medical college, and then those inventories should be used longitudinally for measuring and comparing their depression, anxiety, and stress levels. If their depression, anxiety, and stress levels are found to be high, timely intervention should be provided.

## Abbreviations

KAU: King Abdulaziz University
PSS: Perceived Stress Scale
SA: Saudi Arabia
